# Cue Sources and Cue Utilization Patterns of Social Mentalizing during Two-Person Interactions

**DOI:** 10.3390/jintelligence11090173

**Published:** 2023-08-28

**Authors:** Wenwu Dai, Zhaolan Li, Ning Jia

**Affiliations:** College of Education, Hebei Normal University, Shijiazhuang 050024, China

**Keywords:** first-order mentalizing, second-order mentalizing, social mentalizing, cue sources, cue utilization pattern

## Abstract

Social mentalizing plays a crucial role in two-person interactions. Depending on the target of inference and the content being inferred, social mentalizing primarily exists in two forms: first-order mentalizing and second-order mentalizing. Our research aims to investigate the cue sources and cue utilization patterns of social mentalizing during two-person interactions. Our study created an experimental situation of a two-person interaction and used the “Spot the difference” game to reveal our research question with multi-stage tasks. Our study was divided into two experiments, Experiment 1 and Experiment 2, which examined the cue sources and cue utilization patterns of first- and second-order mentalizing, respectively. The results of the experiments showed that (1) self-performance and other performance are significant cues utilized by individuals during social mentalizing. (2) Individuals employ discrepancies to modulate the relationship between self-performance and first-order mentalizing as well as to adjust the relationship between otherperformance and second-order mentalizing. The results of this study further complement the dual-processing model of mindreading and the anchoring and adjustment hypothesis during social inference.

## 1. Introduction

It is widely acknowledged that humans, as social beings, engage in social interactions that constitute an inherent aspect of life. Quickly inferring the mental states of others is crucial for navigating our social world (Van Overwalle and Baetens 2009). These social inferences, relying on insights on other individuals, are referred to as social mentalizing (Van Overwalle and Vandekerckhove 2013). The process of mentalizing enables individuals to discern and comprehend the goals of others by tapping into what is known as “social intelligence”—the capacity to grasp the thoughts of others as if we could read their minds (Amodio and Frith 2006). Consequently, mentalizing plays a pivotal role in individuals’ social inference, leading to enhanced interaction and communication effectiveness (McDonald and Flanagan 2004). 

In the field of social mentalizing during two-person interactions, the concept of first-order mentalizing, commonly referred to as mindreading, has captivated the interest of numerous researchers as the prevailing form of mentalizing. It primarily involves the inferences individuals make about the mental states of others (Wu et al. 2020). This particular form of social mentalizing underscores the tendency for individuals to adopt a self–other perspective, striving to comprehend whether their thought processes align with those of others during complex social interactions while also seeking to understand the mental states of others (Wu et al. 2020). Within the process of mentalizing, individuals typically derive inferences about their own or another person’s mental state based on various cues. Dai et al. (2017) conducted a common-sense question task to investigate mentalizing mechanisms and found that individuals use both self-performance and other performance to analyze and synthesize information when mindreading. Interestingly, changes in the information on the other party can also affect an individual’s inferences and understanding. Based on their findings, Dai et al. (2017) proposed a dual-process model of mindreading that indicated that individuals rely on two channels of cues in mindreading: self-performance and other performance. One channel infers the other party’s mental state by analyzing the other information provided, while the other channel involves self-simulation to obtain self-performance and to better understand others, which suggests that self-performance and other performance both play key roles in mindreading. In addition to self-performance and other performance, individuals also gain a third significant cue by comparing self-performance and other performance: the discrepancy cue. Wang et al. (2023) introduced a known target agent and investigated whether the discrepancy cue serves as the foundation for mentalizing during social inference. Their findings revealed that, as the discrepancy between the self and others increased, the time taken for participants to provide rank inferences about the target agent also increased, suggesting that discrepancies play a pivotal role as cues when individuals engage in mentalizing. Drawing upon the aforementioned theory and pertinent experimental results, we propose that self-performance, other performance, and discrepancies constitute crucial sources for the cues of individuals in first-order mentalizing, a social mentalizing type, during two-person interactions.

Facing these three cues, how does the individual use the cues? According to the anchoring and adjustment hypothesis, during social inference, individuals establish an anchor and then make adjustments to it in order to make inferences about themselves and others (Epley et al. 2004; Tamir and Mitchell 2010, 2013). Chen and Su (2011) found evidence for a two-stage process in which adult individuals base their mentalizing on their own mental states as an anchor and then correct their egocentrism to make accurate inferences about the mental states of others, ultimately achieving a better understanding of them. This indicates that individuals select the self as an anchor point and adjust for discrepancies accordingly to complete first-order mentalizing. Due to the lack of a real data sample from the participants in the study of their mentalizing mechanisms during social interaction, most mentalizing studies in social reasoning have used self–other discrepancies as the dependent variable and the reaction time to rank others as the independent variable to explore their correlations, which has allowed them to infer that individuals achieve an understanding of others by anchoring themselves and adjusting for differences (Tamir and Mitchell 2013). However, this experimental design can only explore the effectiveness of discrepancies as a cue and cannot directly investigate the pattern or weight of the cues used by individuals in mentalizing. Hence, in this study, we aim to construct an authentic experimental scenario involving two-person interactions to delve deeper into the pattern by which individuals utilize cues while making first-order mentalizing inferences, building upon prior research. Drawing from the aforementioned theories and relevant experimental findings, we propose a hypothesis that discrepancy cues will serve as moderators in the relationship between self-performance, other performance, and first-order mentalizing, respectively.

In the social mentalizing of two-person interactions, not only does first-order mentalizing exist, but second-order mentalizing also plays a key role in the interactions. According to the Interactive Mentalizing Theory (IMT) (Wu et al. 2020), second-order mentalizing concerns individuals’ comprehension of others’ mental states about themselves. When engaging in second-order mentalizing, individuals tend to adopt the other–self perspective, and individuals adjust their confidence levels based on how well their predictions match the observational outcomes to reveal the degree of insight they believe others have into their own mental states. It must be noted that while second-order mentalizing is a new concept in the interactive mentalizing model proposed by Wu et al. (2020), there are few studies that have explored the processing cues on which it relies. Overall, our examination of prior research suggests that individuals mainly rely on discrepancies, self-performance, and other performance when engaging in first-order mentalizing. Having established that both first-order and second-order mentalizing are sub-components of mentalizing and in light of the dual-process model of mindreading, our study suggests that second-order mentalizing and first-order mentalizing may share common cues. If individuals do rely on the same cues during both forms of mentalizing, the question arises as to whether the process of cue utilization and the weight of cue utilization are equivalent in both cases. The anchoring–adjustment hypothesis during social inference and the IMT suggest that differences may exist between the two (Tamir and Mitchell 2013). Specifically, the differing perspectives on which the two types of mentalizing are grounded may mean that the anchoring point used during adjustment varies and that the role played by cues during cue utilization may also differ. Based on this, we hypothesize that self-performance, other performance, and discrepancies are also important sources for cues in the social mentalizing type of second-order mentalizing during two-person interactions, but the pattern of the cues used by individuals in second-order mentalizing may be different from that in first-order mentalizing.

In short, this study is mainly to explore the cue sources and cue utilization patterns of social mentalizing (first-order mentalizing and second-order mentalizing) during two-person interactions. To address these concerns, a two-person interactive experimental situation was created, with the “Spot the difference” game serving as the experimental material, to uncover the cue sources and cue utilization patterns of social mentalizing with multi-stage tasks.

## 2. Methods

### 2.1. Experiment 1: Cue Sources and Cue Utilization Patterns of First-Order Mentalizing

#### 2.1.1. Participants

A total of 48 (24 male) graduate students (age: 20.62 ± 1.88 yrs) participated in this study. All participants were right-handed, with normal or corrected-to-normal vision. They were randomly assigned as pairs; members of pairs were not acquainted with each other before experiment. A total of 24 pairs would then be created, including 8 female–female (F-F) pairs, 8 male–male (M-M) pairs, and 8 female–male (F-M) pairs. Informed consent was obtained from each participant. Participants would receive a fee of 15 yuan after the experiment. The study procedures were approved by the Institutional Review Board of Hebei Normal University.

#### 2.1.2. Tasks and Procedures

**Figure 1:**
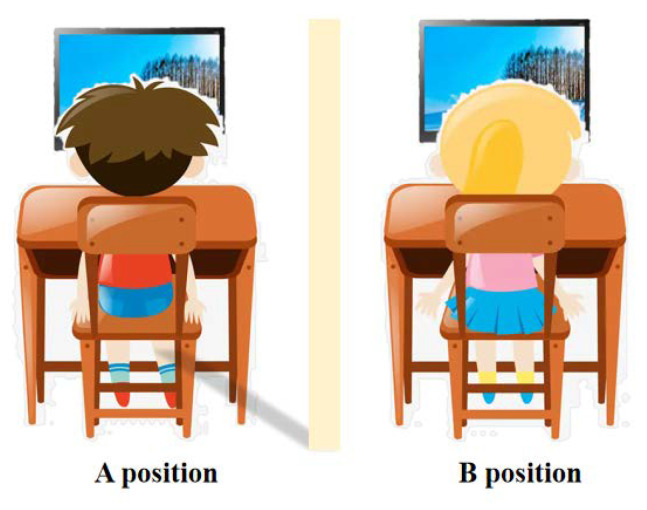
Experimental situation of the two-person interaction.

**Figure 2:**
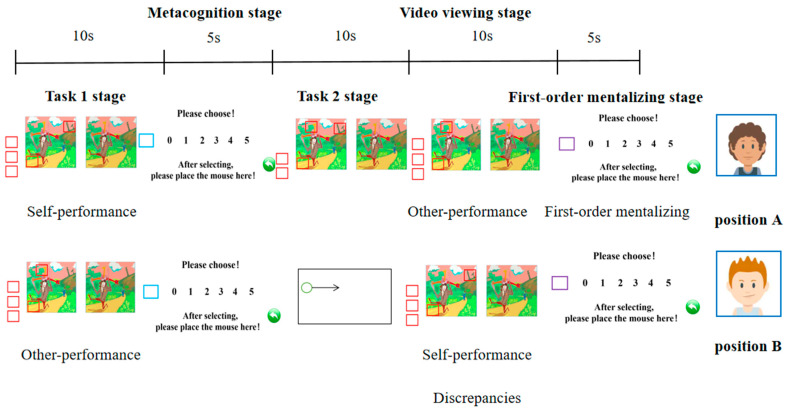
The procedures of experiment 1.

Our experiment designed a scenario for two-person interactions (Figure 1) and implemented the “Spot the difference” game as the experimental task to collect experimental data. The experiment procedure consisted of five stages (Figure 2): task 1, metacognition, task 2, video viewing, and first-order mentalizing judgment. Prior to the formal experiment, the participants were required to complete 2 practice tasks to ensure their full comprehension of the instructions and their ability to carry out accurate operations according to the examiner’s instructions. The formal experiment included 32 trials. 

Task 1 stage: Following the appearance of the “+” sign on the screen, participants were instructed by the examiner to activate the 10 s screen recording function (the recorded video was saved in a shared folder for the other participant to use during the video viewing stage). Two seconds after activating the function, two images with five differences between them were displayed on the screen. Participants were required to drag the red box over the differences they found within ten seconds while the left picture was displayed.

Metacognition stage^1^: participants were required to predict the number of “differences” they would correctly identify within the overall 20 s time frame and to select the corresponding number (0–5) by dragging the blue box.

Task 2 stage: Following the appearance of the “+” sign on the screen, participants continued to complete the task according to the on-screen instructions. Participants located in position A continued to complete the “spot the difference” task for an additional 10 s based on the previously completed Task 1 stage. Participants located in position B needed to drag the mouse according to the random on-screen instructions.

Video viewing stage: Participants followed on-screen prompts to open the respective folder and watched the video of the other participant completing task 1. This video depicted the specific process of the other participant carrying out the task 1 stage, with a duration of 10 s, and it was only allowed to be viewed once. After the video ended, participants closed it and proceeded to the next phase.

First-order mentalizing stage: participants needed to make a first-order mentalizing judgment after watching the video, which involved predicting the number of “differences” the other participant would correctly identify within 20 s and selecting the corresponding number (0–5) by dragging the purple box.

#### 2.1.3. Experimental Variable

Adopting the vantage point of the participant in position A, two experimental variables would be established during the task 1 stage, specifically self-performance and other performance. The former pertained to the accurate count of distinct numbers identified by participant A in the Task 1 stage, whereas the latter constituted the accurate count of distinct numbers identified by participant B. Moving on, the third experimental variable was established during the video viewing stage: discrepancies, also known as the difference in the number of items detected by participants A and B in the Task 1 stage. Based on the nature of these disparities, our study categorized the discrepancies into three distinct classifications: discrepancy_2_, discrepancy_1_, and discrepancy_0_. Among them, discrepancy_2_ represented the situation where participant A found more “differences” than participant B in the Task 1 stage, discrepancy_1_ represented the scenario where participant A and participant B found the same number of “differences”, and discrepancy_0_ represented the situation where participant A found fewer “differences” in the task 1 stage than participant B did. Finally, the last experimental variable was formed in the first-order mentalizing stage: first-order mentalizing, that is, subject A needed to predict the number of “differences” the other participant would correctly identify within 20 s.

#### 2.1.4. Data Analysis

The cue sources and cue utilization patterns of first-order mentalizing based on the perspective of participant A were investigated. Referring to Wang et al. (2023), behavioral data were analyzed using Linear Mixed-Effects Models (LMEM) via lmer function from the lme4 package using R software(4.1.1). Prior to model fitting, using the Q-Q plot to test the normality of the dependent variable, it was found that the dependent variable was normally distributed. All categorical variables were sum coded, and all continuous independent variables were centered at the participant level; extreme data that exceeded ± 3 standard deviations were removed.

According to the dual-processing model of mindreading and the anchoring and adjustment hypothesis, during social inference, our fixed-effects structure included self-performance, other performance, discrepancies, the interaction of discrepancies and self-performance, and the interaction of discrepancies and other performance. Discrepancies were sum coded during model fitting to assess main effects. Our random-effects structure model fitting began with a maximal model (Barr et al. 2013). Random-effects correlation parameters were not included during model fitting. Principal component analysis (PCA) was used in order to determine the variance accounted for by each of the random factors in order to reduce the model to the most parsimonious random-effects structure (Privitera et al. 2022). Models were compared after the removal of each random factor using likelihood ratio tests (LRT) (Privitera et al. 2022). A random factor was only removed if the resultant model was not significantly different from a model that included that variable. Finally, the random effect part of the model was minimized. Our final model in experiment 1 was as follows:

*First-order mentalizing* ~ 1 + self-performance + other performance + discrepancies         + discrepancies × self-performance          + discrepancies × other performance                + (1 + self-performance|participant) + (1|item)

Statistical significance of fixed effects was determined using type III ANOVA test (the *p*-values for the fixed effects were calculated from an F test on Satterthwaite’ s approximation), with the mixed function from afex package. We performed post hoc comparisons with the “Estimated Marginal Means” R package via the emmeans function (Fini et al. 2021).

#### 2.1.5. Results

The above-mentioned final model (R^2^*_c_* = 0.609) was analyzed (Nakagawa and Schielzeth 2013), which yielded a significant main effect of self-performance (*F* (1,479.56) = 10.697, *p* = 0.001), of other performance (*F* (1,525.87) = 33.574, *p* < 0.001), and of discrepancies (*F* (2,692.96) = 7.663, *p* < 0.001). The two-way interaction between self-performance and discrepancies was significant (*F* (2,706.29) = 5.193, *p* = 0.0016); the two-way interaction between other performance and discrepancies was not significant (*F* (2,707.19) = 2.495, *p* = 0.083). Fixed and random effects of the model are shown in Table 1.

**Figure 3:**
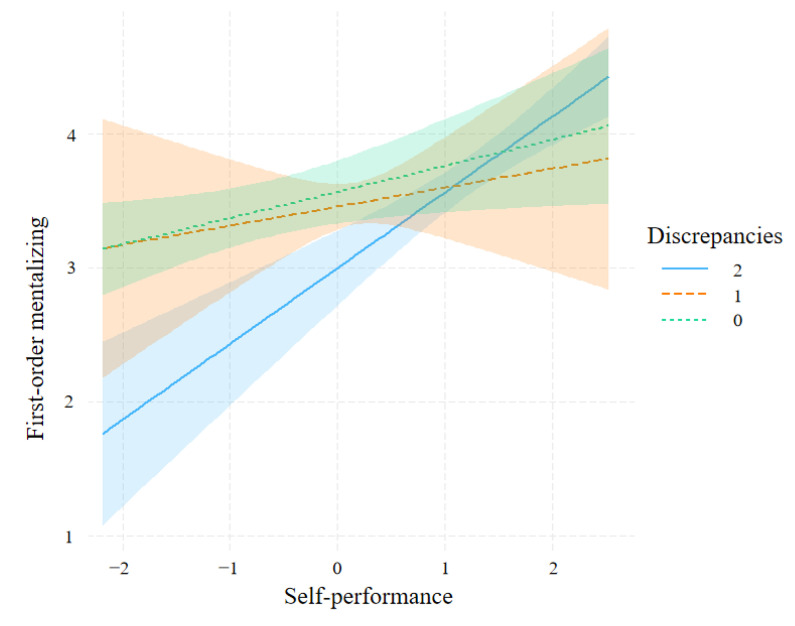
Association between self-performance and first-order mentalizing moderated by discrepancies.

As the interaction was significant, the slope for each level of discrepancies was estimated (Figure 3). Simple slope analysis showed that the slopes of discrepancy_2_ (*b* = 0.56, *p* < 0.001, 95% *CI* = [0.37, 0.76]) and discrepancy_0_ (*b* = 0.19, *p* = 0.02, 95% *CI* = [0.02, 0.37]) were significantly different from zero as a function of self-performance, but that of discrepancy_1_ (*b* = 0.14, *p* = 0.49, 95% *CI* = [−0.26, 0.55]) was not significantly different from zero.

#### 2.1.6. Discussion

Experiment 1 demonstrated that self-performance and other performance were effective cues employed by individuals in performing first-order mentalizing, which aligned with previous research (Dai et al. 2017). According to the dual-process model of mindreading, individuals utilize diverse relevant and available cues to make inferences, where self-performance and other performance are crucial and accessible cues in the process of social interaction. On one hand, leveraging one’s own relevant information for mentalizing is a swift and straightforward heuristic approach that is often adaptive (Gigerenzer and Gaissmaier 2011; Nickerson 2001). One’s own self-centered experiences, such as the degree to which one has completed tasks, tend to reflect other people’s psychological state accurately (Dawes 1989). People frequently infer others’ transient psychological state in a bottom-up manner. On the other hand, since bottom-up information is frequently vague or limited, individuals also use top-down strategies to engage in social reasoning (Mitchell 2009; Nickerson 1999; Royzman et al. 2003). Therefore, while focusing on self-performance, people also incorporate other performance as an important cue into their available information pool.

Experiment 1 also found that discrepancies had a moderating effect on the relationship between self-performance and first-order mentalizing. This suggested that individuals use discrepancies as an adjusting cue to complete adjustment during first-order mentalizing and that self-performance is used as a reference anchor during the adjustment process, which is consistent with previous research (Chen and Su 2011). According to the anchoring–adjustment hypothesis, during social inference, individuals determine the anchor point based on their initial perspective during social interaction and adjust the anchor point based on the self–other difference, ultimately making inferences. As first-order mentalizing starts from the self-perspective, individuals shift from focusing on themselves to representing the psychological state of others, identifying differences between themselves and others, using this discrepancy to adjust the anchor point of self-performance cues, and inferring the psychological state of others (Chen and Su 2011; Epley et al. 2004; Nickerson 1999; Tamir and Mitchell 2010, 2013).

Experiment 1 validated the cues found effective in prior research for first-order mentalizing and also discovered the moderating impact of discrepancies between self-performance and first-order mentalizing, which was a novel finding. While first-order and second-order mentalizing belong to interactive mentalizing processes, differences in perspectives and psychological functions exist between them. Therefore, Experiment 2 continued to utilize behavioral experiments to explore the cue sources and cue utilization patterns of second-order mentalizing and to investigate whether there were commonalities or distinctive differences in cue sources and cue utilization patterns between first-order and second-order mentalizing.

### 2.2. Experiment 2: Cue Sources and Cue Utilization Patterns of Second-Order Mentalizing

#### 2.2.1. Participants

A total of 52 (28 male) graduate students (age: 20.81  ±  1.90 yrs) participated in this study. A total of 26 pairs would then be created, including 10 female–female (F-F) pairs, 8 male–male (M-M) pairs, and 8 female–male (F-M) pairs. Informed consent was obtained from each participant. Participants would receive a fee of 15 yuan after the experiment. 

#### 2.2.2. Tasks and Procedures

The procedure was the same as that in Experiment 1 with one exception, turning the first-order mentalizing stage into the second-order mentalizing stage.

Second-order mentalizing stage: participants needed to make a second-order mentalizing judgment after watching the video, which involved predicting the number of differences that the other thought they would correctly identify within 20 s and selecting the corresponding number (0–5) by dragging the purple box.

#### 2.2.3. Experimental Variable

The variables were the same as that in Experiment 1 with one exception, turning first-order mentalizing into second-order mentalizing. The experimental variable was formed in the second-order mentalizing judgment stage: second-order mentalizing; that is, subject A needed to predict the number of differences that the other thought they would correctly identify within 20 s.

#### 2.2.4. Data Analysis

The data analysis process was the same as that in Experiment 1. In order to conduct a comparative analysis with Experiment 1 and to combine the data analysis results, our final model in the experiment 2 was as follows:

*Second-order mentalizing* ~ 1 + self-performance + other performance + discrepancies         + discrepancies × self-performance          + discrepancies × other performance                + (1 + self-performance|participant) + (1|item)

#### 2.2.5. Results

The above-mentioned final model (R^2^*_c_* = 0.625) was analyzed (Nakagawa and Schielzeth 2013), which yielded a significant main effect of self-performance (*F* (1,540.71) = 19.602 *p* < 0.001) and of other performance (*F* (1,701.40) = 7.923, *p* = 0.005), but there was no significant main effect of discrepancies (*F* (2,750.80) = 2.459, *p* = 0.086). The two-way interaction between other performance and discrepancies was significant (*F* (2,768.61) = 3.096, *p* = 0.046); the two-way interaction between self-performance and discrepancies was not significant (*F* (2,781.80) = 2.257, *p* = 0.105). Fixed and random effects of the model are shown in Table 2.

**Figure 4:**
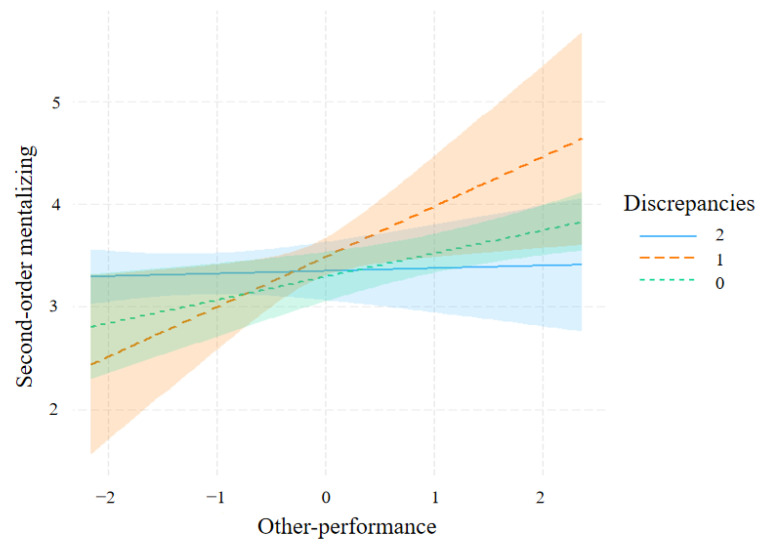
Association between other-performance and second-order mentalizing moderated by discrepancies.

As the interaction was significant, the slope for each level of discrepancies was estimated (Figure 4). Simple slope analysis showed that the slopes of discrepancy_1_ (*b* = 0.49, *p* = 0.02, 95% *CI* = [0.07, 0.90]) and discrepancy_0_ (*b* = 0.23, *p* < 0.001, 95% *CI* = [0.07, 0.38]) were significantly different from zero as a function of other performance, but discrepancy_2_ (*b* = 0.03, *p* = 0.77, 95% *CI* = [−0.15, 0.20]) was not significantly different from zero.

#### 2.2.6. Discussion

The results of Experiment 2 had some similarities to Experiment 1 in that both self-performance and other performance were effective cues used by individuals in performing second-order mentalizing, expanding the applicability of the dual-process model of mindreading. The results of Experiments 1 and 2 demonstrated that the cues used in first-order and second-order mentalizing have some commonalities.

Experiment 2 also found that discrepancies had a moderating effect on the relationship between other performance and second-order mentalizing, which further confirmed the anchoring and adjustment hypothesis during social inference. This result was inconsistent with the result obtained in Experiment 1. The main reason for this difference is that there are differences in the inferential perspectives used in first-order and second-order mentalizing (Wu et al. 2020). The aforementioned discussion mentioned that first-order mentalizing mainly uses the self-perspective to complete mentalizing processing, whereas second-order mentalizing mainly uses the other perspective. Therefore, individuals perform second-order mentalizing by shifting from focusing on themselves to representing the psychological state of others, ultimately inferring what others think about themselves. This indicates that individuals performing second-order mentalizing do not directly use self-performance to complete the process but instead use a simulation processing strategy centered on others (Goldman 1995), using other performance as an anchor and adjusting the anchor point using discrepancies to estimate others’ mental state about themselves.

## 3. General Discussion

These results suggested that individuals use some cues in the two types of social mentalizing during two-person interactions. Specifically, both types of mentalizing use self-performance and other performance in their processing, which is consistent with the dual-process model of mindreading. On the one hand, individuals accumulate psychological knowledge and construct a theoretical system based on this knowledge, and they use this theoretical framework to analyze the cues provided by others’ information to complete mentalizing. On the other hand, individuals also simulate their own or others’ psychological states, imagine similar experiences, activate similar psychological states, and ultimately complete mentalizing. Based on the theory of interactive mentalizing (Wu et al. 2020), individuals typically adopt a self–other mentalizing perspective when engaging in first-order mentalizing. Conversely, a person tends to use a other–self mentalizing perspective when engaging in second-order mentalizing. These findings suggest that various components of mentalizing require different perspectives for predicting targets and making inferences. Because mentalizing is inherently complex, individuals use different cue utilization patterns in the two types of social mentalizing during two-person interactions.

Firstly, when examining the role of cues in various mentalizing processes, differences in behavioral outcomes can be observed. The results from Experiment 1 and 2 indicate that, when engaging in first-order mentalizing, other performance holds greater significance than self-performance (*b*_other performance_ = 0.492, 95% *CI* [0.33, 0.67]; *b*_self-performance_ = 0.300, 95% *CI* [0.12, 0.48]). On the other hand, when engaging in second-order mentalizing, self-performance becomes more critical than other performance (*b*_self-performance_ = 0.407, 95% *CI* [0.22, 0.59]; *b*_other performance_ = 0.247, 95% *CI* [0.07, 0.42]). This shift in emphasis is mainly due to differences in the predicted targets of mentalizing. While first-order mentalizing focuses primarily on inferring others’ mental states, second-order mentalizing centers on inferring others’ beliefs about themselves, thus emphasizing the self and creating disparities in cue weighting.

Second, individuals demonstrate variations in their modulation mechanisms when confronting different mentalizing processes as evidenced by their behavioral results. In the context of first-order mentalizing, individuals primarily utilize discrepancies as a regulating cue in the self-performance and first-order mentalizing relationship. Conversely, in the context of second-order mentalizing, individuals predominantly use discrepancies as a regulating cue in the other performance and second-order mentalizing relationship, primarily due to differences in the underlying perspectives of mentalizing. According to the dual-process model of mindreading (Dai et al. 2017), individuals use simulation-based strategies when engaging in mentalizing. Moreover, the anchoring–adjustment hypothesis during social inference posits that individuals initially process information based on their own mental state when attempting to understand others (Epley et al. 2004; Tamir and Mitchell 2010, 2013). This suggests that, in the context of first-order mentalizing, individuals begin simulating the mental process of others from their own perspective. Conversely, in the context of second-order mentalizing, as an individual attempts to infer others’ thoughts on their own, they start simulating their own mental process from the perspective of the other person. 

This study employed a behavioral experimental approach to probe the cue sources and cue utilization patterns underlying first-order and second-order mentalizing. Through an innovative method, it was found that the cues relied upon by these two types of social mentalizing have common cue sources, while their cue utilization patterns are specific. However, there are still various aspects yet to be explored in future research. Firstly, the experimental context created for this study offered external and cognitive cues to both parties. If the interaction context were to change and if additional cues (such as facial expressions or body language) were to be provided, how would this impact the cue sources and cue utilization patterns of mentalizing between two individuals? Consequently, future research could incorporate cues such as facial expressions (Gonzalez and Chang 2021); speech rhythm (Achim et al. 2013; Gil et al. 2014); body posture (Parkinson et al. 2017); and other aspects in the scenarios of multi-person social interactions, such as two-player game tasks or team cooperation tasks, in order to explore the mechanisms underlying mentalizing between individuals (Singer and Tusche 2014; Wu et al. 2022). Secondly, this study focused heavily on the moderating effect of the discrepancy variable. Previous research has found that, in social reasoning, individuals are influenced by target object similarity when adjusting the discrepancies between themselves and others (Wang et al. 2023). Future research could therefore address the question of whether the number of overlapping regions identified by two individuals in the “Spot the difference” game is another important cue in their interactional mentalizing and how this overlapping cue interacts with other cues in the cue sources and cue utilization patterns of mentalizing. Thirdly, due to certain constraints imposed on the scope of this study regarding two-person social interactions, it was not possible to explore the differences in cue sources and cue utilization patterns underlying spontaneous first-order mentalizing and second-order mentalizing. Consequently, the generalizability of the research findings is somewhat limited. Previous studies have already delved into the processing mechanisms of spontaneous social mentalizing at the individual level (Rice and Redcay 2015). Future research could focus more on spontaneous social mentalizing during two-person interactions and could employ data simulation methods to delve into the topic at a deeper level.

## 4. Conclusions

Our research showed that self-performance and other performance are significant cues utilized by individuals during social mentalizing; individuals employ discrepancies to modulate the relationship between self-performance and first-order mentalizing as well as to adjust the relationship between other performance and second-order mentalizing. The results of this study further complement the dual-processing model of mindreading and the anchoring and adjustment hypothesis during social inference.

## Figures and Tables

**Table 1:** First-order mentalizing through self-performance, other performance, discrepancies, and interactions in the experiment 1 data analysis.

**Fixed Effects**						
**Variable**	***b***	***SE***	***df***	***t***	***p***	**95% *Confidence*** ***Interval* (*CI)***
Intercept	3.338	0.091	57.477	36.596	<0.001	[3.16, 3.52]
Self-performance	0.300	0.092	479.556	3.271	0.001	[0.12, 0.48]
Other performance	0.492	0.085	525.871	5.794	<0.001	[0.33, 0.67]
Discrepancy_1_	−0.340	0.087	662.161	−3.906	<0.001	[−0.51, −0.17]
Discrepancy_2_	0.115	0.056	723.510	2.055	0.040	[0.00, 0.22]
Self-performance × discrepancy_1_	0.264	0.091	725.312	2.891	0.004	[0.05, 0.42]
Self-performance × discrepancy_2_	−0.159	0.136	666.446	−1.168	0.243	[−0.42, 0.11]
Other performance × discrepancy_1_	−0.198	0.091	732.182	−2.171	0.030	[−0.38, −0.02]
Other performance × discrepancy_2_	0.172	0.135	663.361	1.274	0.203	[−0.09, 0.43]
**Random Effects**						
**Cluster**	**Name**		**Variance**		***SD***	
Item	Intercept		0.034		0.184	
Participant	Intercept		0.111		0.333	
	Self-performance		0.019		0.139	
Residual			0.343		0.586	

**Table 2:** Second-order mentalizing through self-performance, other performance, discrepancies, and interactions in the experiment 2 data analysis.

**Fixed Effects**						
**Variable**	***b***	***SE***	***df***	***t***	***p***	**95% *CI***
Intercept	3.376	0.099	61.960	34.111	<0.001	[3.18, 3.57]
Self-performance	0.407	0.092	540.715	4.427	<0.001	[0.22, 0.59]
Other performance	0.247	0.088	701.399	2.815	0.005	[0.07, 0.42]
Discrepancy_1_	−0.028	0.084	718.201	−0.333	0.739	[−0.19, 0.14]
Discrepancy_2_	0.110	0.053	770.065	2.090	0.036	[0.01, 0.21]
Self-performance × discrepancy_1_	0.176	0.089	785.665	1.980	0.048	[0.00, 0.35]
Self-performance × discrepancy_2_	−0.280	0.140	780.839	−2.002	0.045	[−0.55, −0.01]
Other performance × discrepancy_1_	−0.221	0.089	788.286	−2.478	0.013	[−0.39, −0.05]
Other performance × discrepancy_2_	0.241	0.139	778.924	1.735	0.083	[−0.03, 0.51]
**Random Effects**						
**Cluster**	**Name**		**Variance**		***SD***	
Item	Intercept		0.063		0.251	
Participant	Intercept		0.141		0.375	
	Self-performance		0.011		0.107	
Residual			0.313		0.559	

## Data Availability

The data are currently not publicly available due to participant privacy, but they are available from the first author upon reasonable request.

## Note

1 We designed this phase for the following reasons: Wu et al. (2020) proposed that metacognition serves as the foundation for all forms of mentalizing. Thus, before engaging in first-order or second-order mentalizing, individuals need metacognitive processing.
